# Voices from a Multidisciplinary Healthcare Center: Understanding Barriers in Gender-Affirming Care—A Qualitative Exploration

**DOI:** 10.3390/ijerph20146367

**Published:** 2023-07-15

**Authors:** Maeghan B. Ross, Hiba Jahouh, Margriet G. Mullender, Baudewijntje P. C. Kreukels, Tim C. van de Grift

**Affiliations:** 1 Department of Plastic Reconstructive and Hand Surgery, Amsterdam University Medical Centers, Location VUmc, 1081 HV Amsterdam, The Netherlandsm.mullender@amsterdamumc.nl (M.G.M.);; 2 Amsterdam Public Health Institute, 1081 BT Amsterdam, The Netherlands; 3 Department of Medical Psychology, Center of Expertise on Gender Dysphoria, Amsterdam University Medical Centers, Location VUmc, 1081 HV Amsterdam, The Netherlands; 4 Department of Medical Psychology and Psychiatry, Zaans Medisch Centrum, 1502 DV Zaandam, The Netherlands

**Keywords:** transgender, gender-affirming care, barriers

## Abstract

When seeking gender-affirming care, trans* and gender-diverse individuals often describe experiencing barriers. However, a deeper understanding of what constitutes such barriers is generally lacking. The present research sought to better understand the barriers trans* and gender-diverse individuals experienced, and their effects, when seeking gender-affirming care in the Netherlands. Qualitative interviews were conducted with trans* and gender-diverse individuals who sought care at a Dutch multidisciplinary medical center. Twenty-one participants were included, of which 12 identified as (trans) male, six identified as (trans) female, one as trans*, and one as gender-nonconforming (GNC)/non-binary. The interviews were mostly conducted at the homes of the participants and lasted between 55 min and 156 min (mean = 85 min). Following data collection and transcription, the interviews were analyzed using axial coding and thematic analysis. A total of 1361 codes were extracted, which could be classified into four themes describing barriers: lack of continuity: organizational and institutional factors (n_codes_ = 546), patient–staff dynamics (n_codes_ = 480), inadequate information and support (n_codes_ = 210), and lack of autonomy in decision making (n_codes_ = 125). Within our study, trans* and gender-diverse individuals described encountering multiple and diverse barriers when seeking gender-affirming care in the Netherlands. Future studies are needed to evaluate whether individualized care, the decentralization of care, and the use of decision aids can improve the experienced barriers of trans* and gender-diverse individuals seeking gender-affirming care within the Dutch healthcare system.

## 1. Introduction

Research has shown that access to gender-affirming care greatly improves the mental health and overall wellbeing of gender-diverse, transgender, and non-binary individuals [1,2,3,4,5,6,7,8,9]. Encompassing a range of psychosocial, behavioral, and medical interventions, gender-affirming care is designed to “support and affirm” a person’s gender identity when it does not align with the sex assigned at birth (i.e., assigned female at birth, AFAB; assigned male at birth, AMAB) [10,11,12,13,14]. Generally, access to most (insured) gender-affirming care options is dependent upon a classification using the criteria of the Diagnostic and Statistical Manual of Mental Disorders, Fifth Edition (DSM-5), by the American Psychological Association (i.e., “Gender Dysphoria”) or the International Classification of Diseases 11th Revision (ICD-11) by the World Health Organization (“Gender Incongruence”) [15,16]. Not all people who identify as transgender (trans*) and gender diverse experience dysphoria, and although many individuals will have some variation of a social transition (i.e., change of name, pronouns, gender expression, etc.), not all desire and/or have the ability to socially or medically transition (i.e., hormones and surgery) [1,2,3,4].

In recent years, gender-affirming care options have become more accessible, provider education has become more available, and the literature regarding gender-affirming care has grown [17,18,19,20,21]. As a result, many people, mostly non-trans*, have come to believe that access to gender-affirming care is not only more available, knowledgeable, and sensitive, but that the previous barriers trans* and gender-diverse individuals faced when seeking gender-affirming care have been remedied [22,23]. And yet, despite increased access, availability, knowledge, and research, trans* people report experiencing numerous barriers when seeking gender-affirming care, thus indicating that barriers in gender-affirming care persist [24,25,26,27,28,29,30].

In order to understand the concept of barriers in care, there must first be an understanding of an individual’s access to said care. For the purpose of this research, an individual’s access to care is viewed ‘as the possibility to identify health care needs, to seek health care services, to reach the health care resources, to obtain or use health care services and, to actually be offered services appropriate to the needs for care’ [31]. Accordingly, barriers in care can be defined as barriers that result in unmet needs, or delayed, limited, or prevented care [14,31]. Unfortunately, experiencing barriers in care (i.e., accessibility, available services, costs, etc.) is an aspect of seeking healthcare that both cis and trans* individuals face within the current healthcare systems globally [11,24,32,33,34]. Research has shown that in general healthcare settings, trans* individuals are often met, disproportionately to their cis counterparts, with a system that does not adequately meet their needs [35]. Cis individuals do not face the specific additional barriers within trans-specialized clinical settings, including access to gender-affirming psychological, hormonal, and/or surgical care [24,36,37,38].

In an effort to mitigate the number of barriers that individuals report experiencing globally year after year, more and more research studies are being conducted in order to further identify and understand the experienced barriers to care that trans* and gender-diverse individuals face when seeking gender-affirming care. While research into the barriers that trans* individuals face within healthcare is necessary, it is important to conduct this research in a way that avoids becoming “damage research”, or, rather, research that primarily documents the harms experienced by marginalized communities without addressing the underlying causes or effecting change [39]. Well-meaning research methods can inadvertently contribute towards damaging research in trans* studies, leaving individuals with feelings of being “overresearched, and ironically made invisible”.

At present, most studies regarding experienced barriers to gender-affirming care have been primarily conducted in North America and the majority of the reported barriers are contingent on organizational and institutional factors [40,41,42,43,44,45]. Studies have found that the barriers that trans* and gender-diverse individuals experience when seeking gender-affirming care are comprehensive in nature and associated with structural barriers (i.e., waiting lists, insurance coverage, travel time), provider barriers (i.e., insufficient knowledge, gate-keeping, denial of care), ethical dilemmas (i.e., preconceived judgment, referrals, etc.), and care-seeking barriers (i.e., health literacy, rejection of self, etc.) [29,40,42,44,45,46,47,48,49,50]. While the aforementioned barriers have been reported specifically within gender-affirming healthcare settings, these barriers are also experienced by trans* and gender-diverse individuals within general healthcare settings [24].

At the data collection stage of this study, approximately 95% of trans* and gender-diverse individuals seeking gender-affirming care in the Netherlands received their treatment at a specialized gender center in Amsterdam [14]. In a Dutch report, issued by the Netherlands Institute for Social Research, some of the experienced barriers to gender-affirming care that trans* and gender-diverse individuals reported included extensive wait times, lack of psychosocial care, and lack of knowledgeable healthcare professionals [51,52,53]. Generally, the healthcare system in the Netherlands is characterized by universal coverage, mandatory health insurance, and a focus on accessibility, quality, and affordability. The healthcare system involves a combination of public (majority) and private (minority) healthcare providers, with the government ensuring that every resident has access to essential healthcare services through a basic health insurance package. Due to this universal form of healthcare coverage, most of the individual’s transition-related care (i.e., HRT, chest surgery, genital surgery) is reimbursed. However, certain conditions must be met in order to apply for reimbursement for procedures such as fertility preservation, laryngeal prominence reduction, breast augmentation, facial feminization surgery, and voice surgery. The principal barriers that individuals described experiencing in the Netherlands were more focused on access and quality of care than the actual reimbursement of the care itself [53]. In other European countries (i.e., UK, Germany, and Belgium) where most healthcare costs are covered, trans* and gender-diverse individuals seeking gender-affirming care also report the aforementioned barriers [14,54].

A recent survey in the Netherlands found that 67% of adult trans* and gender-diverse individuals have waited more than 18 months for an initial intake and hormone treatment, and another median of 6 months before surgical intake [55]. As of April 2023, there are more than 1400 people on the waiting list for an initial intake appointment at the Vrije University Medical Center (VUmc) gender clinic, with the actual time spent waiting for this appointment being close to three years for adults seeking gender-affirming care [56]. And while barriers in gender-affirming care (i.e., costs and accessibility of qualified providers) are largely dependent on how the healthcare system and gender-affirming care are currently organized, an understanding of the barriers trans* and gender-diverse individuals face within insured specialized multidisciplinary health centers, like in the Netherlands, remains scarce [14,57,58,59,60,61]. Therefore, the aim of this study was not only to identify, but also to gain a better understanding of the barriers in gender-affirming care that led to unmet needs, or delayed, limited, or prevented gender-affirming care for treatment-seeking trans* and gender-diverse individuals in the Netherlands.

## 2. Materials and Methods

### 2.1. Study Procedure

The qualitative data obtained in this pilot study formed part of a larger research project with the objective of developing a patient-reported outcome measure based on the experiences of trans* individuals [62]. The larger study involved collecting qualitative data on various aspects of individuals’ healthcare experiences. Additionally, two secondary analyses were conducted using the sample from this dataset [63,64]. Approval for the study was granted by the Ethical Review Board of the Amsterdam University Medical Center (UMC), VuMC (no. 2017.617), and all participants provided both written and oral informed consent.

### 2.2. Study Setting

At the gender center, the gender team includes various departments and medical professions. The team consists of healthcare providers from fields such as psychology, psychiatry, endocrinology (hormone specialists), gynecology, and plastic surgery. Additionally, urology, dermatology, ENT (Ear, Nose, and Throat), speech therapy, surgery, and facial surgery are also involved based on individual treatment wishes.

Access to the facilities was granted upon referral by a general practitioner. Generally, individuals began with consultations with a psychologist, where they discussed their experiences of gender incongruence, their needs, and the available options for gender-affirming interventions. The diagnostic procedure typically lasted 6–8 months with monthly visits. The process concluded with a multidisciplinary team evaluating the individual’s eligibility for gender-affirming care. While not all individuals desire a hormonal transition, hormonal treatment follows the diagnostic phase. If individuals had already started hormone treatment somewhere else, treatment would be continued with potential adjustments. During gender-affirming hormone treatment, individuals had appointments every 3 months with an endocrinologist and a psychologist. On average, most individuals will have had at least 12 months of hormone replacement therapy before qualifying for gender-affirming surgeries.

Public funding covered diagnostics, hormonal therapy, breast removal, and genital surgery, while other surgeries were not included. Facial hair removal (up to 10 appointments), psychotherapy, and speech therapy were covered, and facial surgery was only covered in exceptional cases. At the time, the Amsterdam clinic followed the Diagnostic and Statistical Manual of Mental Disorders, fourth edition, text revision (DSM IV-TR) classification system, and until 2014, sterilization was required for legal gender change. Since the 2014 change in legal requirements, more flexibility in individual treatment pathways has occurred.

### 2.3. Participants and Recruitment

Eligible individuals were aged 16 years or older, had received a diagnosis of “gender dysphoria”, had initiated or completed the process for gender-affirming procedures in the Netherlands, were fluent in Dutch or English, and were capable of providing informed consent. The research team or their healthcare providers from the Amsterdam University Medical Center, peer support groups, or social media approached eligible participants for recruitment. Purposeful sampling was employed to ensure a diverse range of participants in terms of age, gender, and healthcare history. Recruitment took place over a three-month period (August 2018 to October 2018). Participation was voluntary, and it was emphasized that their involvement would not influence their future care. All participants in this study had either received or expressed a desire to receive gender-affirming surgery. Each participant received a EUR 50 gift card as a token of appreciation, which they were informed about after their participation in the research.

### 2.4. Data Collection

The in-person, one-on-one interviews were conducted by the first author (M.R.), a non-binary-identifying psychologist and trained interviewer with previous experience in qualitative research. The majority of the interviews took place at the participants’ homes and had an average duration of 85 min, ranging from 55 min to 156 min.

A semi-structured interview guide was utilized, covering a wide range of topics such as healthcare experiences, procedure outcomes, recovery, mental health, and body image. This guide allowed participants to provide detailed accounts of their experiences and perceptions of the current healthcare system [62]. The interview topics were based on existing literature concerning gender-affirming care and trans* healthcare [62]. Five specific questions explored the barriers encountered in accessing gender-affirming care: (1) Could you describe your initial experience seeking professional help for your gender identity? (2) Have you faced any obstacles in obtaining gender-affirming care? (3) How would you characterize the individuals who have provided care for you? (4) What kind of information and advice were you given by healthcare providers? (5) How do you think healthcare professionals could improve the quality of care provided to you?

The participants had the freedom to pause or terminate the interview at any point and were also able to withdraw from the study during or after the completion of the interview. All participants completed the interview in a single session, and no participant chose to withdraw or provide comments or corrections on the interview transcripts.

### 2.5. Data Extraction and Analysis

All interviews were audio-recorded and transcribed verbatim. Subsequently, for use in the larger project, the transcripts were translated into English and anonymized by removing personal identifiers [62]. For the purpose of this study, the data were subjected to line-by-line coding using interpretative codes by a coding team consisting of two researchers (M.R. and H.J.). All codes were cross-verified by a third member of the research team (T.G.). Quotes specifically related to barriers that resulted in unmet needs or hindered, delayed, or limited access to gender-affirming care were extracted from the data and imported into a Microsoft Excel spreadsheet for further analysis.

Following the approach outlined by Braun and Clarke, thematic analysis was employed to analyze the data [65]. Initially, a set of codes was generated through axial coding to identify potential themes and subthemes. The coders reviewed and discussed all the identified themes until a consensus was reached. The remaining codes were then utilized to internally validate and refine the themes and subthemes. Similar to the first set of potential themes, the final set of themes, definitions, and possible biases were extensively discussed among the authors until a consensus was achieved. Finally, all findings were thoroughly reviewed, and illustrative quotes were selected to represent each theme.

### 2.6. Reflexivity Statements

As a queer, non-binary individual who identifies within the LGBTQAI+ community, M.R. acknowledges that their personal experiences and identities may have influenced their understanding and interpretation of the data. Being a part of the queer community, M.R. can bring valuable insights and perspectives into the research process, enabling a more nuanced analysis of the findings. However, it is important to acknowledge that their personal experiences might introduce biases that could impact the interpretation of the data. To mitigate this, M.R. took measures to reflect upon their own positionality and biases throughout the research process, engaging in an ongoing self-reflection and seeking feedback from colleagues to ensure rigor and diverse perspectives in the analysis.

T.G., a cisgender, gay man, in addition to supervising this qualitative study, helped with the thematic analysis of the data. While T.G. does not share the same lived experience as trans* individuals, his experiences as a member of the LGBTQAI+ community may have influenced his understanding and interpretation of the data. To address this, T.G. actively engaged in critical self-reflection, sought diverse perspectives, and engaged in discussions with his colleagues throughout the analysis to ensure the findings were accurately represented. T.G. also endeavored to approach the data with openness, empathy, and a commitment to understanding the unique experiences and challenges faced by trans* individuals seeking care.

As a heterosexual female medical student, H.J. played a role in the thematic analysis for this qualitative study. Her personal background and sexual orientation may differ from the experiences of trans* individuals accessing healthcare. It is essential to acknowledge that her own perspectives and biases could have influenced the analysis process. To address this potential bias, H.J. actively engaged in critical self-reflection and sought input from diverse voices throughout the analysis process. H.J. approached the data with an open mind, a commitment to understanding the unique experiences of trans* individuals, and a dedication to representing their voices accurately.

## 3. Results

### 3.1. Participant Characteristics

A total of 57 trans* and gender-diverse individuals were invited to take part in this study; 21 responded and consented to participate (response rate  =  37%). The reasons for not responding to the invitation were not recorded. The participant characteristics are displayed in Table 1. The age of the participants ranged from 18 to 62 years with a mean age of 39 years (SD = 15). Of the 21 participants, 20 participants had started hormone replacement therapy (HRT) and 14 participants had undergone gender-affirming surgical procedures. 

### 3.2. Extracted Codes and Resulting Themes

A total of 1361 codes were extracted, which were classified into four themes describing barriers: lack of continuity: organizational and institutional factors (n_codes_ = 546), patient–staff dynamics (n_codes_ = 480), inadequate information and support (n_codes_ = 210), and lack of autonomy in decision making (n_codes_ = 125).

### 3.3. Theme 1: Lack of Continuity: Organizational and Institutional Factors

Continuity in healthcare refers to the coordination and integration of care services, ensuring that care seekers receive comprehensive and continuous care over time. Barriers in continuity can be related to institutional and/or organizational factors.

The waiting time individuals experienced throughout the transition phases was another of the most discussed barriers within gender-affirming care. The participants reported having to wait excessive amounts of time for the intake appointment, the diagnostic phase, and the gender-affirming care that followed. The participants addressed the (potential) impact the waiting lists and wait times had on their psychological wellbeing, and also described feeling frustrated and as if they were not moving forward within their transition.

*“Because for them it’s ‘Oh you just have to wait a year, you know it’s really a thing that you really have to put a lot of thought into.’ For me, it’s just…I was ready the day that I found out and I started preparing everything… by the end of the month, I was already living as a man at work, at school, and in my private life, and then having to go a year and a half without hormones. It’s so humiliating… Humiliating. It is so bad.” (Participant 01, male/trans male, HRT, chest surgery (mastectomy), genital surgery (hysterectomy, bilateral salpingo-oophorectomy, colpectomy)*

Participants described feeling, within the current healthcare system of the Netherlands, as though there was little room to customize their transition. As a result, the participants described experiencing a system that was unable to accommodate their individual needs and, thus, care preferences. Additionally, some participants objected to a standard trajectory, citing that it is “a limited view on care for trans* people because not everyone experiences dysphoria in the same way”.

*“I think that I would like to see such an organization where they are not trying to put you in a certain box of you are this, but that there is actually a kind of openness in every step. Also, in the order in which you want to do things. For example, I know a couple of pioneers at the VU because they first had chest surgery and now, they go to the hormones. That is a pretty special order of things and I think that it is very important that those things can be done.”(Participant 19, gender queer/GNC/non-binary, age 27, none)*

The participants suggested that certain protocols and policies were not beneficial to trans* individuals seeking care. Within gender-affirming care, this is mainly related to the mandatory waiting time and the conditions required to gain access to gender-affirming care (i.e., social transitioning). Oftentimes, additional trans* protocols and policies were non-existent within the organizations and departments at the hospital that are not specialized in gender-affirming care. Many participants described situations where cisgender-oriented protocols and policies were inadequate in ensuring trans-appropriate care, and some participants noticed that the health professionals of these departments were more likely to misgender participants or treat them differently.

*“I had to have an echo and I had previously agreed with my doctor that we would do this via my stomach. Once I got to the echo place they refused to do it via my stomach, so I left. Where is the communication then? Where is the connection between the departments?” (Participant 08, female, age 56, HRT, genital surgery (vaginoplasty))*

Ultimately, the participants described the aforementioned institutional and systemic barriers as weighing heavily upon not only their trust (or lack thereof), but also their feelings of safety within the hospital setting. The participants described that such experiences only continued to perpetuate their feelings of being misunderstood and undersupported when seeking gender-affirming care.

### 3.4. Theme 2: Patient–Staff Dynamics

The participants described often experiencing a lack of knowledge and understanding from their healthcare professional(s) regarding their gender identity and desired gender-affirming care choices. The experiences described ranged from mental healthcare professionals within the specialized gender-affirming care being unfamiliar with non-cisgender identities to physicians specialized in gender-affirming care invalidating certain trans* identities by maintaining a binary perception of how transitioning should look.

*“…the psychologist didn’t take me serious at all. They were like okay, this is a thing…a fetish or something, you know? And I felt like I was totally not taken seriously then…” (Participant 11, female, age 23, puberty inhibitors, HRT)*

*“The surgeon came to me afterward, and totally didn’t have any understanding of it all. They said: Now you have to watch out with tampons. I think: Do you really think I put a tampon in there?” (Participant 06, male, HRT, chest surgery, genital surgery (hysterectomy, bilateral salpingo-oophorectomy, metoidioplasty with urethral lengthening, phalloplasty with urethral lengthening, erectile prosthetic, testicle implants))*

Oftentimes, the participants described feeling like they needed to educate their mental healthcare provider regarding their gender identity and subsequent healthcare wishes. Some described experiencing that, even when their mental healthcare professional within the specialized gender-affirming care was familiar with *“gender dysphoria”* and various gender identities, they did not necessarily have the proper knowledge, training, or experience to best serve them.

*“They are helpful and everyone tries to empathize, but it is as if you are trying to explain to someone who has never been in love what being in love is. Everyone hears you and everyone is polite to you, but nobody really gets it.” (Participant 17, female, age 36, HRT)*

*“I think that a lot of people who have worked for years for [organization], that they still look through the glasses of the 80s or 90s. And continue to use the same procedures and thought processes. I don’t know if that is still appropriate.” (Participant 17, female, age 36, HRT)*

Generally, the participants described that when approaching gender-affirming care, they were already anticipating experiencing barriers in care and, thus, they described having a reluctance to seek care, a fear of being denied care, and anxiety in seeking care. Oftentimes, the inappropriate care that the individuals described experiencing was due to a lack of understanding and training among healthcare providers. For example, a lack of appropriate accommodations played a major role for AFAB participants who had gynecological procedures—often placed in the same space as cis-identifying females with cis norm protocols.

*“I had to check in at the obstetrics and gynecology ward. And you could just hear this record scratch when I arrived. I had to be there a day before the procedure and I just felt so small, I felt humiliated and I felt like everything I had achieved up until that point was gone in an instant. I know it’s their specialty, but I just wish I had had a bed on the traumatology ward or something.” (Participant 16, male, HRT, chest surgery (mastectomy), genital surgery (hysterectomy, bilateral salpingo-oophorectomy))*

Some participants also described being less likely to disclose how they really felt with regard to their gender identity for fear of being denied access to gender-affirming care. As a result, the participants, if they were able to, would search elsewhere for a different mental healthcare professional with more knowledge and/or experience.

In order to improve care, the participants emphasized that healthcare professionals should be better educated on the various gender identities, so that providers can then “understand it better, that they take it seriously.” The participants specifically emphasized that healthcare professionals should be better trained in providing gender-affirming care.

*“I just think that it is important that all kinds of different professionals in that area around that gender team are also aware of all those different identities. I do not mean knowing 100 million gender identities and pronouns that exist or something like that, but more, for example, that it is important to know the difference between, people who identify themselves as agender or people who identify themselves as gender fluids because it can simply have different kinds of consequences.” (Participant 19, gender queer/GNC/non-binary, age 27, none)*

### 3.5. Theme 3: Inadequate Information and Support

When asked about how participants had received information, the opinions of the participants about the quality and the quantity of the information varied. Among the participants who described being well informed, some individuals added that they would have liked to receive more information regarding fertility care and the trajectory of their transition at the beginning of their transition process. Those participants who described being poorly informed reported that the information provided about topics such as the psychosocial aspects of transitioning, sexuality, the physical effects of HRT, and alternative healthcare professionals was insufficient.

In several instances, participants described needing help coming to terms with their gender identity and/or care option(s) for “gender dysphoria” before even considering gender-affirming care. Some participants described that when they tried to seek such care at gender-affirming care centers, they were denied that care without a further recommendation for psychological care elsewhere. They described experiencing that there was a lack of transparency in the care options and what care was or was not available, and why.

*“[Fully transitioning] was the only thing they could help me with, they said: ‘Yes, you can go through that process now.’ But at that time, I didn’t dare. Because back then I knew that if I had to do that, I had to tell my school everything, I had to tell my family everything, I hadn’t told my family at all, I hadn’t told anyone at school, not even my friends. So, I didn’t like that at all. I thought it was way too scary… way too big of a step for me. And then they actually said at the VUmc, ‘Well, if you don’t get the gender-affirming treatment, then don’t.’ It was either do it or don’t. So, then I didn’t and then I was gone. And then I thought, well, that was the only place I could go for my problem. And they couldn’t help me.” (Participant 14, female, age 28, HRT).*

Despite the fact that the participants had routine check-ups with mental healthcare professionals throughout the beginning of their transition, many participants described experiencing a lack of psychological support overall. Most prominently, the participants described a lack of support regarding the psychological impact that different aspects of gender-affirming care can have upon the individual. For example, the participants described experiencing a lack of guidance regarding the physical effect of the HRT both during and following their transition. Of the participants who had received gender-affirming surgeries, all reported that the mental health check-ups ceased post-operatively.

*“Yes, you’re guided relatively intensely psychologically and medically before you transition, but during the transition there’s relatively little guidance. The surgeries had a lot of impact for me and I had some good guidance throughout, but because I sought it out myself. It isn’t standard practice, and it should be. Medically, you’re sent home, but without proper explanation. You don’t really know what to expect. That could be improved upon.” (Participant 01, male/trans male, HRT, chest surgery (mastectomy), genital surgery (hysterectomy, bilateral salpingo-oophorectomy, colpectomy))*

Another important aspect of seeking gender-affirming care is the individual’s social transition. Coming out within the individual’s social environment is a major component for both trans* individuals who do and do not wish to medically transition. Many participants described being uncertain about how to ‘come out’ to different groups within their social environment. Participants described being unaware of the societal expectations and implications of the gender role typically associated with their gender identity. Due to a lack of guidance, they felt ill-equipped to meet these expectations and cope with the resulting implications.

*“There is no explanation like… ‘You are going to look more masculine. Remember that you look more threatening to people, keep that in mind.’ There is no guidance for transgender women… ‘Remember if a gentleman offers something to drink then he wants something from you. You look different.’ There is no guidance in there.” (Participant 15, male, age 41, HRT, chest surgery, genital surgery (hysterectomy, bilateral salpingo-oophorectomy, colpectomy, metoidioplasty))*

Moreover, the participants described encountering situations where providers contradicted one another, exaggerated the negative aspects of gender-affirming care, or even shared false information.

*“Every time I would come from one of those particularly heavy appointments I would just come back and just tell my friends I have no idea what she told me and this bad thing could happen or maybe I am not even able to get the surgeries for this or vague reasons that she didn’t know how to explain and it’s not true when I actually get there. I get that they are trying to manage your expectations but it is HARSH…and it is weird because it’s not…it is health care…so to exaggerate the negative consequences so much…why? Its… it just felt really…there were some dark days there where I really had to think well I have to do this because the alternative I can’t bear…so I have to keep going, even if it’s terrible…but now that I am actually there…and it has been okay.” (Participant 09, male/trans man, age 26, HRT, chest surgery)*

Also, participants expressed a need for the provision of more timely and comprehensive information, as well as peer discussion groups and more descriptive reviews of the procedures and post-operative reality.

*“Like I would have wanted to hear more clearly: now we’re going to do hormones for a year and then you can be prepared for the laser treatment, but then you have to apply for it in time. If they had said that all a little more clearly, and also about what was expected of me, it would have been easier for me.” (Participant 14, female, age 28, HRT).*

*“I really missed hearing other people’s experiences. What I did need was their opinions on the changes; which surgeries they had, and especially about how they felt about the results.” (Participant 06, male, HRT, chest surgery, genital surgery (hysterectomy, bilateral salpingo-oophorectomy, metoidioplasty with urethral lengthening, phalloplasty with urethral lengthening, erectile prosthetic, testicle implants))*

Inadequate information and support left many participants with suboptimal guidance throughout the duration of their transition, especially post-operatively and once their transition trajectory was completed. The participants recommended providing better support during the social transition phase, as well as more thorough aftercare regarding post-operative sex following genital surgeries.

*“To move around in the world after a transition is sometimes more than just the physical change and possible speech and voice corrections. Sometimes movement therapy and/or behavioral lessons are needed to make someone more feminine. Also, dos and dont’s around the use of makeup hair, and how to dress (at least that is if you want to blend in in today’s women’s world, there are of course always exceptions). It is good to point out things to prospective women that they should pay attention to because many of them do not see it for themselves and nobody points out them. Like moving and presentation of them/themselves.” (Participant 18, female, HRT, chest surgery (breast augmentation), FFS, chondrolaryngoplasty, genital surgery (vaginoplasty), voice surgery (glottoplasty))*

### 3.6. Theme 4: Lack of Autonomy in Decision Making

Within the decision-making process, the barriers individuals described experiencing were most often related to (1) the concept of gatekeeping and (2) a lack of autonomy. The participants described that experienced gatekeeping was one of the major barriers in seeking and obtaining gender-affirming care. Most participants described how access to gender-affirming care felt entirely dependent on the decision of their healthcare professional, even when they had given informed consent to proceed with their transition.

*“Yea, it is [the gender team’s] choice…and it isn’t even the choice of the psychologist you talk to—it is the choice of the whole gender team. They have never even seen me.” (Participant 11, female, age 23, puberty inhibitors, HRT)*

The participants experiencing their healthcare provider(s) as a gatekeeper presented three key concerns. First, the participants reported being extremely aware of the control healthcare professionals had over their access to gender-affirming care. Therefore, not only did most participants describe being very careful about how they would present themselves to their healthcare professional, but they also described being hesitant to disclose personal information that might jeopardize their access to said gender-affirming care. Secondly, many participants described experiencing that they had to explain and defend not only their choices, but themselves, and, ultimately, their bodies to their healthcare professionals.

*“But I did feel like … I would say they [mental health care professionals] have this just black and white thing that you have to be transgender enough to get the care and that…that felt really bad as well.” (Participant 09, male/trans man, 26, HRT, chest surgery)*

Thirdly, the participants felt that they had no choice but to accept the conditions of their healthcare professional and healthcare system in order to gain access to gender-affirming care, even if they did not agree with their providers’ point of view. One participant described feeling that, despite feeling like nothing was wrong with him, he had to agree that something was “wrong” with him in order to receive the care he needed.

*“There is nothing wrong with me. But she kept hammering a bit on it…so then I felt I had to agree in order to get the care I needed.” (Participant 20, male, age 27, HRT, chest surgery)*

Additionally, the participants also reported experiencing that healthcare professionals often disregarded their individual needs and care preferences when making care decisions. As a result, the participants described feeling excluded from the decision-making process.

*“…the fact that someone decides what your identity should be because it’s technically easier is pretty insane. So, I think it’s important that they know what kind of impact that can have. They didn’t really talk about that; it was more indirect than that.” (Participant 01, male/trans male, HRT, chest surgery (mastectomy), genital surgery (hysterectomy, bilateral salpingo-oophorectomy, colpectomy))*

Even when participants had the opportunity to make an autonomous decision, they described experiencing that they were either not made aware of alternative care options, or that they experienced being guided towards a certain care option, thus inhibiting the participant’s self-determination and further ability to gain access to desired and appropriate forms of care.

*“I have had a lot of discussions directing me towards the colpectomy and I felt like the plastic surgeon was trying to guide me towards certain care pathways. There are still standard forms of care and I know that some things are surgically easier to perform and result in fewer complications, but I think it can be very important for someone to choose an operation with more complications if the results suit their wishes more.” (Participant 01, male/trans male, HRT, chest surgery (mastectomy), genital surgery (hysterectomy, bilateral salpingo-oophorectomy, colpectomy))*

Several participants felt as though their provider’s decision regarding treatment options weighed more heavily than their own treatment wishes, ultimately leading the participants to feel as though they needed to agree with the provider’s treatment pathway even if it was not fully what they wanted. The participants suggested including the individuals seeking care in the decision-making process by acknowledging their individual needs and care preferences, as an attempt to remedy the aforementioned barriers.

## 4. Discussion

This explorative study identified some of the experienced barriers in care that trans* and gender-diverse individuals face when seeking gender-affirming care in the Netherlands. It was the first in-depth study of barriers in an insured specialized multidisciplinary healthcare setting. Four themes emerged from the thematic analysis: lack of continuity: organizational and institutional factors, patient–staff dynamics, inadequate information and support, and lack of autonomy in decision making. Experiencing one, or all, of these barriers can impact an individual’s mental and physical health, as well as their trust in the healthcare system in general. These barriers will subsequently be discussed in the light of the larger transition gender-affirming care provision and its providers that individuals are going through.

### 4.1. Centralization of Care and Subsequent Waiting Times

When seeking care within insured specialized multidisciplinary centralized health centers, as in the study setting, insured care generally requires adherence to standardized protocols and regulations. For example, in the Netherlands, insured care requires a specific diagnosis based on the Diagnose Behandel Combinatie (DBC) (diagnosis treatment combination) in which the amount of reimbursement for treatments are predefined. In theory, standardized protocols allow for more consistent and predictable care delivery [66]. However, in the context of gender-affirming care, standardized protocols within centralized clinics can also limit access to gender-affirming care for trans* and gender-diverse individuals that may deviate from “traditional” or rather binary, heteronormative, and cisgender-based medical frameworks [18,25,46,67]. While the centralization of care can enhance expertise, improve cross-functional communication, and enhance the overall quality of care, it can also increase bureaucracy, induce bottlenecks, and limit individuals’ agency and flexibility [68,69,70]. In our study, we found that the extensive waiting time was a major barrier, a finding that was consistent with previous research [31,42,51,53,63,71,72]. The participants in the current study described that these waiting times had, or could have, led to delayed, inappropriate, or inadequate care, ultimately impacting long-term health outcomes, patient satisfaction, and system costs. For these barriers, these participants recommended the decentralization of gender-affirming care because it allows trans* individuals to seek care at other organizations or by health professionals with shorter wait times, a finding also in line with previous research [14,72]. However, despite the fact that the Netherlands is currently in the middle of a major effort toward the decentralization of gender-affirming care, this unfortunately has not yet led to a decrease in the current waiting list for access to gender-affirming care [71,73].

### 4.2. Power (im)Balance in Decision Making: The Relationship between Provider and Care Seeker

The (im)balance of power between healthcare professionals and individuals seeking gender-affirming care was an important topic during the interviews. Consistent with the current literature, the participants of this study described how they experienced an imbalance of power in three main ways. First, within the current decision-making model, healthcare professionals have the ability to grant or deny access to gender-affirming care, thus, from the participants’ perspective, taking on the role of the ‘gatekeeper’ [50,61,74,75,76,77,78,79]. The fact that the healthcare providers are required to establish a diagnosis (i.e., DBCs) for someone to be entitled to coverage of healthcare costs may also be directly related to gatekeeping. Second, the focus of healthcare professionals on healthcare outcomes rather than ‘patient’ satisfaction may lead to the subsequent experienced disregard of the participants’ needs and care preferences [80,81,82]. An example of this could be that surgeons tend to be more focused on surgical outcomes and aesthetics according to cisgender norms, rather than the individual’s opinion of the outcome—concepts that are rarely in agreement with one another [83]. And third, the autonomy of the participant is restricted because the professionals determine readiness for, or access to, gender-affirming treatments (i.e., genital or chest surgeries) [61,84,85,86]. Oftentimes, the participants in our study described feeling that they needed to conform to the healthcare preferences and beliefs of their healthcare professional regarding “gender dysphoria” and gender identity in order to obtain access to desired forms of gender-affirming care, which was also described in previous research [87,88]. To reduce the power imbalance, the participants described that a more collaborative decision-making model is desired.

In order to facilitate decision making, access to evidence-based and comprehensible information about gender-affirming care is required. While the concept of informed consent is based on the principle of respect for one’s autonomy, shared decision making is a synergistic process between the individual seeking care and the healthcare professional, which involves introducing choices and goals, comparing alternatives, and discussing decision-making roles [89,90]. Despite how nice this sounds, the reality is often much more intricate. Systemic barriers remain a very real issue and can hinder providers in terms of offering true patient-centered care (i.e., lack of education, resources, and societal attitudes) [24,27,40,91]. As was seen in our research, the participants described feeling marginalized, disrespected, or dehumanized when providers were unable to offer true patient-centered care. Comparable to other studies, the participants in our study described wanting to be involved in the decision-making process and feel as though there were actually decisions to be made regarding their care options [7,79,92,93]. And while providers generally agree that shared decision making is a general best practice [94], previous research highlights that sometimes, shared decision making may lead to moral distress when healthcare providers feel it is not in line with (their assessment of) the client’s best interest (i.e., duty of non-maleficence). This is a topic often under-discussed with clients and could further perpetuate feelings of “gatekeeping” [86,95]. Previous research has shown that the use of decision aids led to increased knowledge, more accurate risk perceptions, a greater number of decisions consistent with patients’ values, a reduced level of internal decisional conflict for patients, and fewer patients remaining passive or undecided [96]. And yet, our findings indicate that participants struggle to access true shared decision-making avenues when seeking gender-affirming care. Specifically, the participants described the inadequate information and support during and after transition as a significant barrier to gender-affirming care [53,57]. The participants also described having difficulty accessing qualified and knowledgeable health professionals [47,48,53,97,98]. And while decision aids, in recent years, have been used more often within gender-affirming care (i.e., genderaid.org), in order for shared decision making to actually be effective in gender-affirming care, an equal partnership between the health professional and the individual seeking care must be established—including involvement and encouragement to participate in the decision-making process (i.e., genderaid.org) [7,95,99,100,101].

### 4.3. Role of Cis and Heteronormative Frameworks in Difficulties in Changing Approaches to Gender-Affirming Care

The concept of the gender binary—allocating individuals into two categories, (trans)male and (trans)female—assumes that gender identity, expression, and role inherently align with one of the two. This concept itself excludes the possibility of trans identities outside the gender binary, such as non-binary, genderfluid, and genderqueer. This assumption also suggests that individuals who identify within a specific gender identity all have similar needs, healthcare wishes, and preferences (i.e., to pass as their desired gender). However, previous studies have shown that Dutch trans* and gender-diverse individuals seeking gender-affirming care identify within a range of non-cisgender identities and that the needs and care preferences of these individuals cannot be solely determined based on their gender identity [12,53,102,103,104]. Subsequently, protocols and policies externalizing the gender binary, such as a standard healthcare trajectory and obligatory wait time for gender-affirming surgeries, only contribute to the further institutional erasure of trans* needs and identities.

Historically, medical and societal norms were based on a binary, heteronormative, cisgender framework that led to a “narrow” model for trans* care (and legislation) [105], which, as a result, perpetuated transphobia and discrimination through the use of pathologization and discrimination within healthcare [105]. In this study, the participants described that they experienced needing to act “trans* enough” in order to obtain desired access to gender-affirming care. In turn, this can conflict with the individual’s autonomy and care preferences, creating feelings of invalidation, inequity, and mistrust in the healthcare system—which can lead to negative health outcomes, such as delays in care (i.e., hormonal and surgical interventions) and worsened mental health [63,73,106]. Despite the fact that the concept of gender is moving away from binary categorical norms and is viewed more on a continuum, the majority of institutional protocols and policies have yet to adapt accordingly. As a result, the lack of policies that accommodate trans identities and trans bodies in the current healthcare system can contribute to the individual experiencing barriers within gender-affirming care [58].

In recent years there has been a growing recognition of the importance of taking into account local and cultural contexts when providing gender-affirming care. This recognition acknowledges that the needs and experiences of trans* individuals can vary across different cultures, communities, and regions [94]. As a result, there has been an effort to make guidelines less prescriptive and more flexible and adaptable to diverse cultural and local contexts [94]. However, guidelines still need to provide evidence-based recommendations and standards to ensure the safety and wellbeing of trans* individuals. Finding the right balance between flexibility and standardization remains a challenge for organizations (i.e., WPATH) [94]. Further research regarding the adaptation of guidelines specific to gender-affirming care is necessary.

### 4.4. Strengths and Limitations

There were several strengths to this study. While previous research has identified barriers to gender affirming-care in a Dutch specialized multidisciplinary healthcare setting, this was the first study to qualitatively assess specific barriers. The sample participants were diverse in their gender-affirming care options and age range, although some participants had received care before 2014 and were thus subjected to a more binary and protocolled form of care (i.e., until 2014, individuals seeking gender-affirming care were required by law to undergo sterilization if they wanted to change gender in legal documents). The interviews were conducted with a queer-identifying psychologist trained in qualitative interviewing and took place mostly at individuals’ homes, or where they felt the safest. Although there were strengths to this study, some limitations may be noted. The method of participant recruitment and selection may have introduced selection bias into this study [39,107,108]. In particular, it is worth commenting that the individuals were approached by members of the study team, and in some cases, that was linked with the VUmc. Employing community-based research methods and participation would have increased the quality of this research. Furthermore, individuals who were receiving or already had received gender-affirming care at the VUmc in Amsterdam were approached to participate in this study. Therefore, individuals who were unable to access gender-affirming care were not included. As a result, additional access barriers may not have been identified. Furthermore, unique barriers in gender-affirming care specific to further marginalized subgroups may not have been identified. Also, our findings present the barriers to care that trans* individuals reported experiencing within the Dutch healthcare system and could potentially be perceived as damage research. However, this study did address some of the barriers identified in previous research, and thus aimed to improve the overall wellbeing and access to care for trans* individuals. Additionally, we have provided recommendations that emphasized the need to challenge and disrupt the dominant damaging narrative all together. Moreover, because the data were translated prior to coding, a chance of bias/loss of meaning within the coded dataset is possible. Further research is recommended in order to explore additional access barriers and barriers that may be specific to subgroups regarding participant ethnicity, socio-economic status, education level, and gender identity.

## 5. Conclusions

Trans* individuals experience barriers to gender-affirming care, some of which are also experienced in non-specialized gender-affirming care. Reducing these barriers requires implementing and locally adapting guidelines, and further shaping shared decision making in this field. The current study provides tools to target this, specifically focusing on adapting the organization of care to reduce waiting times, increasing empowerment of trans* individuals, and support for shared decision-making models. Additionally, recommendations are given regarding education encompassing more inclusive norms and values, and increased gender sensitivity.

To improve trans* research, it is important to center the voices of trans* individuals, prioritize participatory approaches, and uphold ethical considerations. This shift can promote social justice and positive change for trans* communities. Actively engaging policymakers, healthcare providers, and stakeholders through dissemination, collaboration, advocacy, education, and dialogue are ways in which research findings can be effectively translated into meaningful policy reforms, practice improvements, and interventions that address and overcome the barriers faced by trans* individuals.

Future studies are needed to evaluate whether trans-inclusive protocols, the decentralization of care, and the use of decision aids can reduce the barriers experienced by individuals seeking gender-affirming care within the Dutch healthcare system. 

## Figures and Tables

**Table 1:** Participant characteristics.

ID	Age	Gender Identity	Sex Assigned at Birth	Gender-Affirming Procedures	Further Procedure Wishes
1	27	Male/Trans male	AFAB	HRT, chest surgery (mastectomy), genital surgery (hysterectomy, bilateral salpingo-oophorectomy, colpectomy)	Genital surgery (phalloplasty)
2	52	Female	AMAB	HRT, genital surgery (vaginoplasty), chondrolaryngoplasty	Chest surgery (breast augmentation)
3	62	Male/Trans man	AFAB	HRT, chest surgery (mastectomy), genital surgery (hysterectomy, bilateral salpingo-oophorectomy, metoidioplasty)	
4	18	Male/Trans man	AFAB	HRT	Chest surgery (mastectomy), genital surgery (hysterectomy, colpectomy, phalloplasty, testicle implants, prosthetic)
5	40	Male	AFAB	HRT, chest surgery (mastectomy), genital surgery (hysterectomy)	
6	60	Male	AFAB	HRT, chest surgery (mastectomy), genital surgery (hysterectomy, bilateral salpingo-oophorectomy, metoidioplasty with urethral lengthening, phalloplasty with urethral lengthening, erectile prosthetic, testicle implants)	Genital surgery revisions (correct prosthetic)
7	37	Male	AFAB	HRT, chest surgery (mastectomy), genital surgery (hysterectomy, colpectomy, bilateral salpingo-oophorectomy)	Genital surgery (phalloplasty)
8	56	Female	AMAB	HRT, genital surgery (vaginoplasty)	Genital surgery (vaginoplasty corrections)
9	26	Male/Trans man	AFAB	HRT, chest surgery (mastectomy)	Genital surgery (hysterectomy, phalloplasty)
10	58	Transgender	AMAB	HRT	Chest surgery (breast augmentation)
11	23	Female	AMAB	HRT, puberty inhibitors	Genital surgery (vaginoplasty)
12	21	Female	AMAB	HRT, puberty inhibitors, genital surgery (orchidectomy)	Genital surgery (intestinal vaginoplasty)
13	52	Male	AFAB	HRT	Chest surgery (mastectomy), genital surgery (hysterectomy)
14	28	Female	AMAB	HRT	Genital surgery (vaginoplasty)
15	41	Male	AFAB	HRT, chest surgery (mastectomy), genital surgery (hysterectomy, bilateral salpingo-oophorectomy, colpectomy, metoidioplasty)	
16	22	Male	AFAB	HRT, chest surgery (mastectomy), genital surgery (hysterectomy, bilateral salpingo-oophorectomy)	Genital surgery (colpectomy, phalloplasty)
17	36	Female	AMAB	HRT	Genital surgery (vaginoplasty), chest surgery (breast augmentation), FFS
18	61	Female	AMAB	HRT, chest surgery (breast augmentation), FFS, chondrolaryngoplasty, genital surgery (vaginoplasty), voice surgery (glottoplasty)	
19	27	Gender queer/GNC/non-binary	AFAB	None	Chest surgery (mastectomy)
20	27	Male	AFAB	HRT, chest surgery (mastectomy)	Genital surgery (hysterectomy)
21	26	Male/Trans male	AFAB	HRT, chest surgery (mastectomy)	Genital surgery (hysterectomy, phalloplasty)

AFAB: assigned female at birth, AMAB: assigned male at birth, HRT: hormonal replacement therapy, FFS: facial feminization surgery, GNC: gender non-conforming. Reprinted with permission from Ross et al. [64] (2023, Ross).

## Data Availability

Interview data is not available due to confidentiality, privacy and ethical concerns.
